# Current approach and novel perspectives in nasopharyngeal carcinoma: the role of targeting proteasome dysregulation as a molecular landmark in nasopharyngeal cancer

**DOI:** 10.1186/s13046-021-02010-9

**Published:** 2021-06-21

**Authors:** Ramon Yarza, Mateo Bover, Maria Teresa Agulló-Ortuño, Lara Carmen Iglesias-Docampo

**Affiliations:** 1 Medical Oncology Division, Hospital Universitarioss 12 de Octubre, Avda. Córdoba s/n, E-28041 Madrid, Spain; 2 Clinical and Translational Laboratory, Instituto de Investigación Hospital 12 de Octubre (I+12), Madrid, Spain; 3 Lung Cancer Group, Clinical Research Program (H12O-CNIO), Centro Nacional de Investigaciones Oncológicas (CNIO), Madrid, Spain; 4 Biomedical Research Networking Centre: Oncology (CIBERONC), Instituto de Salud Carlos III, Madrid, Spain; 5 Facultad de Fisioterapia y Enfermería, Universidad de Castilla La Mancha (UCLM), Toledo, Spain

**Keywords:** Nasopharyngeal carcinoma, Proteasome, Bortezomib, Immunotherapy, Epstein Barr virus

## Abstract

Nasopharyngeal carcinoma (NPC) represents a molecularly paradigmatic tumor given the complex diversity of environmental as well as host dependent factors that are closely implicated in tissue transformation and carcinogenesis. Epstein Barr Virus (EBV) plays a key role in tissue invasion, hyperplasia and malignant transformation. Therefore, EBV related oncoviral proteins such as Latent Membrane Protein family (LMP1, LMP2), Epstein Barr Nuclear Antigen 1 (EBNA1) and EBV related glycoprotein B (gB) are responsible for inducing intracellular signalling aberrations leading to sustained proliferation and further acquisition of NPC related invasive nature and metastatic potential.

Dysregulation of proteasome signaling seems to be centrally implicated in oncoviral protein stabilization as well as in modulating tumor microenvironment. Different studies in vitro and in vivo suggest a potential role of proteasome inhibitors in the therapeutic setting of NPC. Furthermore, alterations affecting proteasome signalling in NPC have been associated to tumor growth and invasion, distant metastasis, immune exclusion and resistance as well as to clinical poor prognosis. So on, recent studies have shown the efficacy of immunotherapy as a suitable therapeutic approach to NPC. Nevertheless, novel strategies seem to look for combinatorial regimens aiming to potentiate immune recognition as well as to restore both primary and acquired immune resistance.

In this work, our goal is to thoroughly review the molecular implications of proteasome dysregulation in the molecular pathogenesis of NPC, together with their direct relationship with EBV related oncoviral proteins and their role in promoting immune evasion and resistance. We also aim to hypothesize about the feasibility of the use of proteasome inhibitors as part of immunotherapy-including combinatorial regimens for their potential role in reversing immune resistance and favouring tumor recognition and eventual tumor death.

## Background

Nasopharyngeal carcinoma (NPC) is an Epstein-Barr virus (EBV) associated malignancy arising from the nasopharynx. It displays remarkable characteristics regarding ethnic and geographic distribution, including higher prevalence in Southeast Asia, South China, Northern Africa, Greenland and Alaska [1–3].

**Fig. 1:**
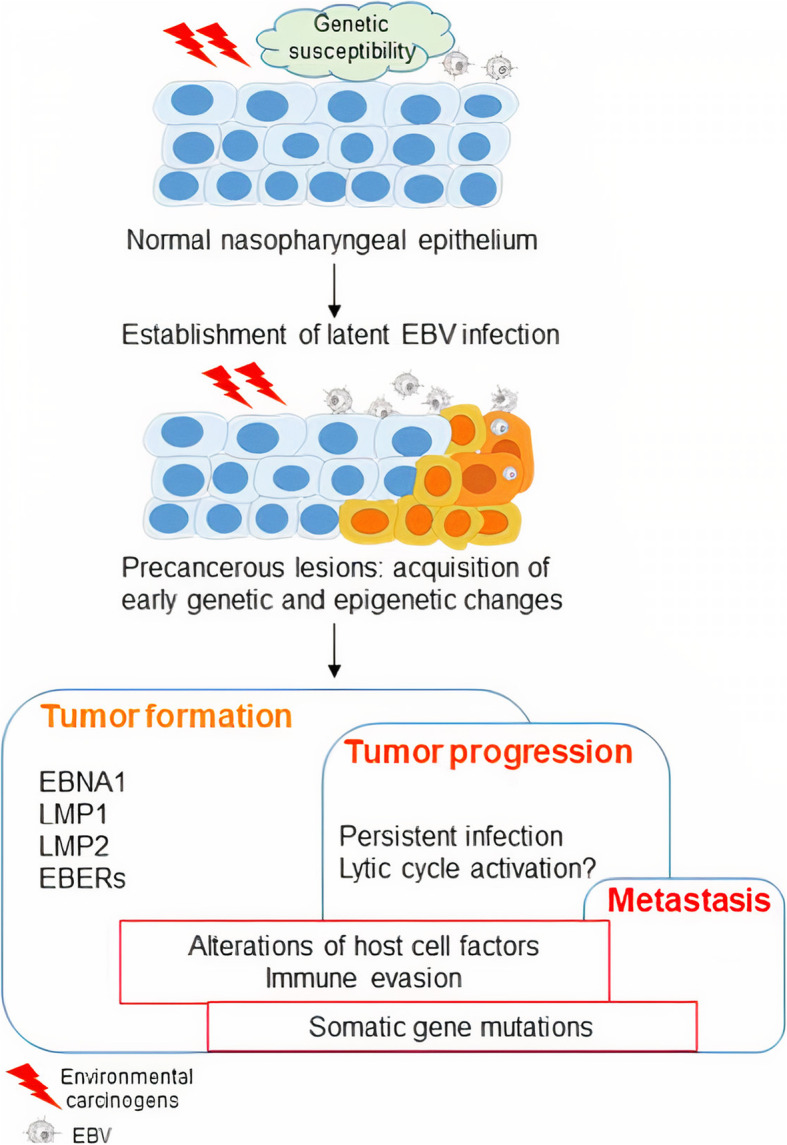
Diagram illustrating EBV associated nasopharyngeal damage and eventual carcinogenic transformation of NPC

Malignant transformation and tumor progression of NPCs are directly related to environmental factors, lifestyle, dietary habits, latent EBV infection, local inflammation, host immunity, and genetic and epigenetic alterations [4–10] as briefly summarized in Fig. 1. A thorough understanding of these processes is therefore important so as to contribute to further development of suitable therapeutic strategies.

A combined modality of concurrent chemo-radiotherapy based on platinum regimen is the main approach for locally advanced NPC, showing an improvement in overall survival, although there is a relatively high chance of recurrence in non-metastatic NPC [11–16]. Given the strong relation between EBV infection and NPC, plasma EBV DNA detection has been used for screening among susceptible population helping the diagnosis at earlier stages [17–19].

EBV is a human gamma-herpes virus which establishes lifelong asymptomatic infections in more than 90% of individuals worldwide [20–22]. This virus can adopt a latent phase or a lytic phase, being capable to switch from latent to lytic replication in epithelial cells to replenish the virus reservoir [23]. The contribution of EBV to oncogenesis has been a subject of study in several tumors, including NPC, and usually associated with poor prognosis [24]. Latent EBV resides inside tumor cells and it is associated with a restricted pattern of viral gene expression and therefore only a subset of latent viral proteins are expressed in EBV-positive NPCs [25, 26]. Until now, latent EBV infection was believed to be involved in tumorigenesis and the lytic reactivation of EBV infection was related to cell death. However, recent studies are claiming that both latent and lytic EBV genes can contribute to carcinogenesis of NPC, as the latent and lytic proteins can maintain the proliferation and survival of EBV positive cancer cells by deregulation of both cell cycle and proapoptotic pathways [27].

Four major types of EBV latent infections have been identified. NPC exhibits frequently the type II latency infection characterized by expressing several non-coding RNAs such as EBERs, LMP1, LMP2, BARTs and EBNA viral proteins [28–30]. On the other hand, experimental strategies targeting EBV-associated tumors involve upregulation of viral lytic gene expression showing that Unfolded Protein Response (UPR) leads to EBV lytic gene expression [31]. Moreover, induction of EBV lytic cycle could lead to apoptosis of EBV-positive NPC cells [32] although these approaches harbor the inconveniences of virion production, which could otherwise contribute to tumorigenesis [33].

Proteasomes are responsible for the degradation of ubiquitinated proteins, and its role has been extensively documented in EBV-positive NPCs. Ubiquitin system is responsible for protein processing and degradation. In this sense, targeted proteins are primarily poly-ubiquitinated and eventually degraded by the 26S subunit. This process is mediated by consecutive activation of several enzymes, namely ubiquitin-activating enzyme (E1), ubiquitin-conjugating enzyme (E2) and ubiquitin-protein ligase (E3), which may exert protein poly-ubiquitination, enabling further proteasome recognition and eventual proteolysis [34–36].

Cancer cells are highly dependent on proteasome-mediated degradation because of their intrinsically accelerated tumor turn-over. Thus, therapeutic efforts have been directed so as to promote direct proteasome inhibition which may in turn facilitate intracellular protein accumulation. An excess of intracellular misfolded protein-burden leads to further endoplasmic reticulum stress and cellular death. EBV induced proteasome truncation seems to be centrally implicated in viral replication as well as in EBV-induced carcinogenesis [37]. Consequently, proteasome inhibition-targeting drugs are being extensively studied in diverse human neoplasms, including NPC, among which bortezomib, SAHA, carfilzomib and romidepsin are to be mentioned (Table 1).

**Table 1:** Table summarizes main basic studies assessing the role of proteasome inhibitors in NPC tissue samples, cell lines, xenografts and murine models

Study	Cell lines	Patient Samples / animal studies	Treatment	Endpoints or objectives	Outcomes/Results
Li et al. [36]	CNE1, CNE-LMP1+	**–**	MG-132	Study of LMP1 regulating the expression of MDM2	MDM2 expression is upregulated by LMP1 through a post-ubiquitination mechanism.
Hau et al. [38]	HONE1, HONE1-EBV, CNE2-EBV C666–1, NP460-hTERT	**–**	MG-132	Examination of LMP1 protein levels in different EBV-infected cell lines	- LMP1 protein is rapidly degraded via proteasome-mediated proteolysis. - Binding of Id1 to LMP1 suppressed polyubiquitination of LMP1 and may be involved in stabilization of LMP1 in EBV-infected nasopharyngeal epithelial cells. -Proteasome inhibitor could effectively stabilize LMP1 protein in EBV-infected cells - Id1 could interact and stabilize LMP1 by suppressing LMP1 polyubiquitination.
Gainullin et al. [39]	CNE1, CNE2, TWO3, HONE1	9 NPC tissues 10 normal epithelial tissues from chronic nasopharyngitis patients	MG-132	Role of LMP2A in the accumulation of cofilin in NPC.	- LMP2A was found to interfere with cofilin degradation in NPC cells by accelerating the proteasomal degradation of Cbl and Syk. - Interference with cofilin degradation may enhance the metastatic potential of NPC cells
Zhou et al. [40]	HONE1/Akata, HK1/Akata, C666–1, CNE1/Akata, CNE1 (EBV-)	43 NPC tissues PBMCs BALB/c nude mice	Triptolide MG-132	Anti-cancer effect of triptolide and mechanism of EBNA1 in NPC cells	Low dose of triptolide reduce the half-life of EBNA1 and significantly decreased EBNA1 expression by promoting the process of proteasome ubiquitin pathway.
Zhang et al. [22]	CNE1, CNE2, HNE1, HONE1, HK1, SUNE1, C666–1, NPEC2-Tert, NPEC5-Tert Primary NPC cell lines	–	–	Interplay between EBV gB and host proteins	- FBXO2 ubiquitinates and degrades glycosylated gB. - Identification of SCF^FBXO2^ as an E3 ubiquitin ligase targeting EBV envelope protein gB.
Meng et al. [41]	CNE1, CNE2 (S18, S26), SUNE-1 (5–8)	50 NPC tissues	MG132	Molecular mechanisms of NPC metastasis	- S100A14 promoted the ubiquitin-proteasome-mediated degradation ofIRAK1 to suppress NPC cellular migration. - Lower S100A14 expression was significantly correlated with shorter patient OS and DMFS.
Pan et al. [42]	CNE1, CNE2, HONE1, C666.1	45 NPC tissues 30 control	LLnL MG132 LLM	Functional relationship between Jab1 and p27 protein expression	- Jab1-mediated p27 degradation in a proteasome-dependent manner. - Overexpression of Jab1 correlated with poor survival in NPC patients.
Liu et al. [43]	HONE1 HK1	–	Curcumin MG-132	Effect of curcumin on the proliferation, cycle arrest, and apoptosis of EBV^+^ NPC cells	Curcumin induced EBNA1 degradation via the proteasome-ubiquitin pathway
Friboulet et al. [44]	C666–1 CNE2 (EBV-)	13 NPC biopsies C15 and C17 (EBV^+^ in nude mice) NPC xenografts	MG132 Epoxomicin	Functions of c-IAP2 in NPC cells	- RMT 5265 induces the proteasome-mediated degradation of c-IAP2, resulting from the enhanced polyubiquitination of c-IAP2
Hui et al. [45]	HONE1, HK1-EBV, HONE1-EBV, HA, C666–1	Female BALB/c nude (nu/nu) mice	Bortezomib, SAHA (Vorinostat)	Mechanisms of apoptosis and effects on lytic cycle activation of EBV	- Combination of bortezomib and SAHA synergistically induce killing of a panel of NPC cell lines and suppresses the growth of NPC xenografts in nude mice. - Bortezomib inhibits SAHA’s induction of EBV replication and abrogates production of infectious viral particles in NPC cells.
Xu C. et al. [46]	NP69, CNE2, Hone1, C666–1		MG132	Mechanisms by which EBV elude immune responses	- LMP1 inhibits Sendai virus mediated type I interferon production and downregulates RIG-I signaling pathway by promotion RIG-I degradation dependent on proteasome. - EBV employs a unique strategy to evade RIG-I mediated immune responses.
Jiang et al. [44]	CNE2, CNE1		Bortezomib (PS-341)	Mechanism by which immune evasion affects the response to treatment of NPC.	- Bortezomib downregulates IFNγ-induced IDO expression via inhibition of JAK/STAT1 signaling pathway. - Bortezomib can promote IkB-α phosphorylation-ubiquitination to release NF-kB from IkB-α.

Given the importance of EBV related oncogenesis in NPC as well as the extensively acknowledged role of proteasome associated tumor transformation with further implications in neoplastic proliferation and perpetuation, our aim with this review is to thoroughly assess the molecular basis and oncologic implications of the ubiquitin-proteasome system in NPC as well as its therapeutic significance so far. This may further broaden the clinical and molecular perspective of the rationale beyond the suitability of targeting the ubiquitin-proteasome pathway, both as single therapy and as part of novel combinatorial strategies in the management of NPC.

### The role of EBV related viral proteins in proteasome dysregulation and their implications in NPC carcinogenesis

NPC is molecularly characterized by EBV mediated type II latent infection. In this scenario, the main genetic expression related to the presence of latent EBV infection is composed by *LMP1* (latent membrane protein 1), *LMP2A* (latent membrane protein 2A), *LMP2B* (latent membrane protein 2B), *EBNA1* (Epstein Barr nuclear antigen 1), *BARF1* (Epstein–Barr virus BamHI-A rightward frame 1), as well as two small nuclear RNA namely EBERs (EBV-encoded small RNAs), and the heterogeneously spliced group of BART RNAs [38].

LMP1 is a transmembrane protein which belongs to the tumor necrosis factor receptor (TNFR) family and is constitutively activated in EBV infected cells leading to further activation of multiple downstream signaling pathways in a ligand-independent manner. Even if variable levels of LMP1 protein may be detected in NPC, incidence of tissue LMP1 mRNA detection has been reported in up to 90% of all cases published so far, which supports its central role in the pathomolecular development of NPC [47]. However, regulation of LMP1 levels in NPC is poorly understood. Several cellular host factor and intracellular signaling events could also modulate LMP1 expression, although results described are, in some cases, inconclusive [48–53]. Different studies suggest that LMP1 protein is rapidly degraded via proteasome-mediated proteolysis in physiologic conditions [38, 54]. In this sense, overexpression of Id1 (inhibitor of DNA binding/differentiation 1) seems to further stabilize LPM1 protein in EBV-infected cells. So on, LMP1 has been described to upregulate the expression of Id1, which in turn, leads to increased formation of Id1-LMP1 complexes. These phenomena are able to consequently suppress protein ubiquitination, thereby stabilizing LMP1 in EBV-infected nasopharyngeal epithelial cells [38]. As a consequence, proteasome inhibitors may effectively stabilize this protein in EBV-positive NPCs.

On the other hand, overexpression of TP53, which is widely known to be responsible for cell cycle regulation, is often found in NPC, even though established mutations of TP53 are infrequent in this tumor. Unlike other double-stranded DNA oncoviruses, EBV does not inactivate TP53, however different studies have demonstrated the role of LMP1 which contributes to consecutive activation of NF-kB and AP-1 signaling which leads to further p53 accumulation [25, 37, 55]. Interestingly, Li et al. demonstrated that somatic alterations concerning the NF-kB signaling may occur in a LMP1-independet manner, suggesting that both, LMP1 mediated NF-kB alterations as well as independently occurring somatic NF-kB aberrations may dually contribute to molecular pathogenesis of NPC [56].

In addition to the aforementioned, overexpression of the protooncogene MDM2 has also been widely reported in NPC samples, and its presence has been associated to both EBV infection as well as to tumor metastasis. Besides MDM2 self-ubiquitination, LMP1 has been also described to further increase MDM2 expression by directly regulating the MDM2 ubiquitination. Such phenomenon could be explained as MDM2 ubiquitination occurs in a different linkage manner which shows different functions rather than single degradative signaling via proteasome pathway, which indicated its crucial role in the oncogenic signaling transduction [36]. As a consequence, MDM2 overexpression promotes the rapid degradation of p53 through enhanced proteasome-dependent signaling [57, 58]. Thus, MDM2 could be further regulated by LMP1 through a post-ubiquitination occurring mechanism, and represents an important way for eventual MDM2 burdening in the carcinogenesis of NPC. In this way, LMP1 is thought to play a central role in the carcinogenesis of NPC.

Another EBV related oncoviral protein is Latent Membrane protein 2 A (LMP2A) which has been commonly detected in EBV-positive NPC cells *in vivo* [59]. In vitro studies have also shown that LMP2A promotes survival of tumor precursor cells by facilitating a migratory phenotypic switch on epithelial cells [39, 60]. In addition, cofilin, an actin depolymerizing factor, has also been found to be overexpressed in NPC samples [61], and its degradation is proteasome-mediated. Since the association of viral LMP2A and cofilin results in cofilin stabilization, mainly due to dysregulation of ubiquitin-mediated cofilin turnover in tumor cells, it has been hypothesized that this connection might lead to increased cellular motility contributing to tumor dissemination [61].

As mentioned above, EBNA1 (Epstein Barr nuclear antigen 1) represents another EBV-associated key protein which has been shown to be responsible for the regulation of viral DNA synthesis as well as for episome segregation during mitosis [62]. Triptolide, a natural compound with the pharmacologic capacity of further inhibiting proliferation of EBV positive NPC cells by inducing EBNA1 degradation in a caspase 9 dependent manner [40], was deeply studied by Zhou and cols. Who demonstrated that triptolide may increase transcription levels of EBNA1 while decreasing total protein production in NPC. Moreover, the authors showed that treating NPC cells with MG-132 increased EBNA1 expression, suggesting that EBNA1 degrades through proteasome-ubiquitin pathway [40]. Therefore, triptolide seems to promote EBNA1 instability and eventual degradation through a proteasome-ubiquitin related pathway. In addition, EBNA1 has also been described to be centrally implicated in the modulation of several signaling cascades including TGF-β, STAT1, and NF-kB pathways, among others. Furthermore, EBNA1 also reduces p53 levels by interacting with the ubiquitin specific protease USP7, avoiding the deubiquitination and further stabilization of p53 [41].

**Fig. 2:**
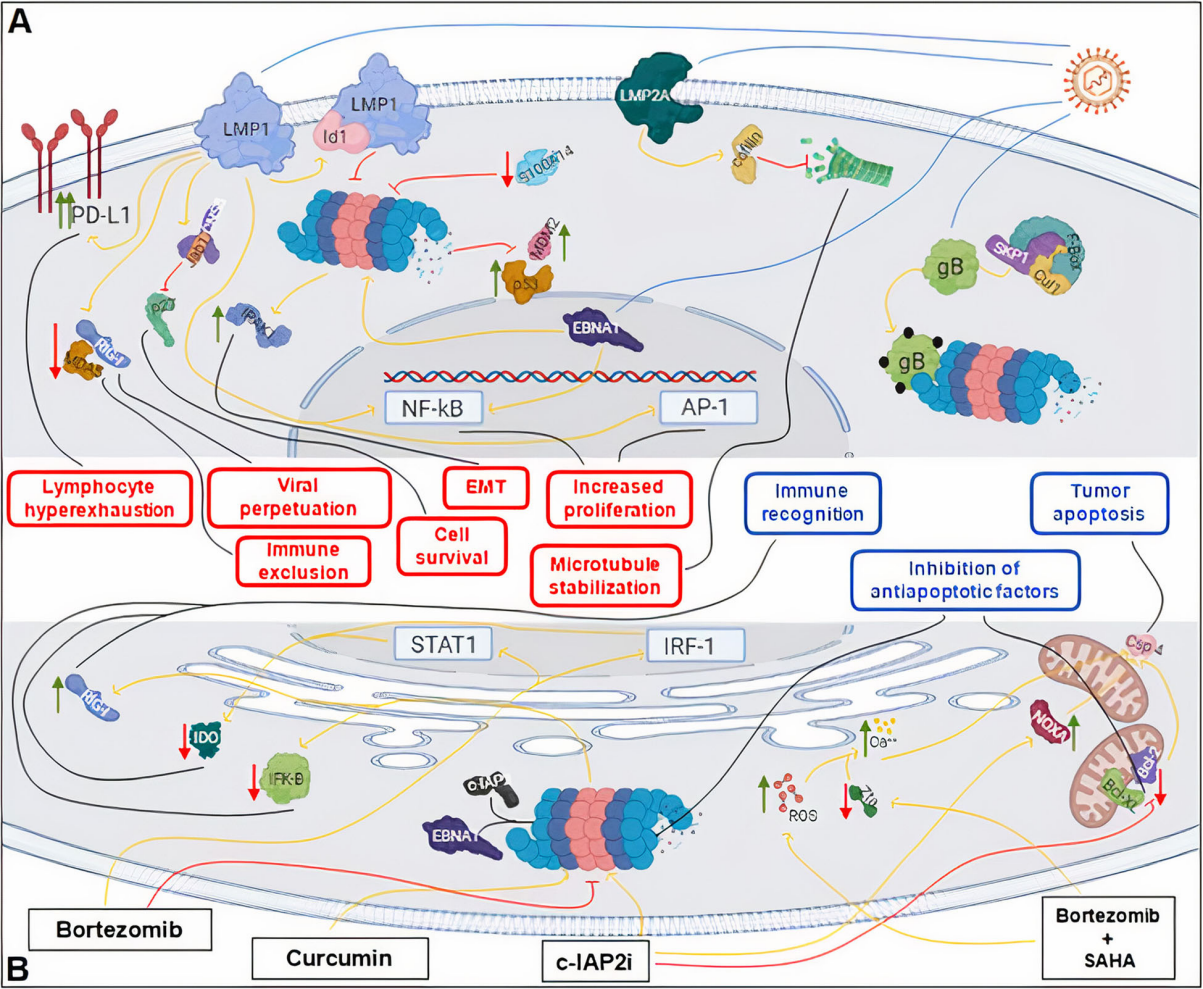
Figure represents metabolic implications of EBV and proteasome dysregulation in NPC. Green arrows illustrate intracellular activation pathways. Red arrows show intracellular inhibitory pathways. Blue and black lines highlight initial and conclusive points of the pathways. **A** Biosignaling pathways associated to EBV infection and proteasome dysregulation in NPC cells. **B** Therapeutic implications of proteasome inhibitors in the molecular pathogenesis of NPC. **FBXO2 accounts for multimeric SKP1/Cul1/F-box

On the other hand, EBV related glycoprotein B (gB) is expressed in almost all herpesvirus. This protein is a core component of the viral fusion machinery and plays an important role in viral entry intro epithelial cells by promoting cell-to-cell fusion. This envelope protein is modified by the addition of high-mannose-linked N-glycans, and it has been shown that F-box only protein 2 (FBXO2), an SKP1/Cul1/F-box protein glycan-dependent E3 ubiquitin ligase substrate adaptor attenuate EBV infectivity by targeting N-glycosylated gB via ubiquitin-proteasome pathway [22]. Therefore, since FBXO2 promotes gB ubiquitination, proteasome inhibitors may prevent FBXO2-mediated degradation of gB. Molecular signaling pathways as well as EBV related aberrations are summarized in Fig. 2.

So on, the role of proteasome in the setting of cellular stability and proteic machinery seems to establish a *continuum* in which there is a viral-mediated basal cellular functioning that accounts for different activity rates of intracellular signaling pathways modulated by oncoviral proteins which in turn leads to further instability of physiologic proteasome functioning. Despite the fact that proteasome inhibition might play a role in further stabilization of oncoviral proteins and, therefore, in perpetuating cellular transformation, an established *continuum* remarks that proteasome does not display a basal physiologic role in tumor cells. Far from that, there is an unbalanced activity of proteasome which further favors oncoviral protein stabilization and activity. Therefore, whole inhibition of ubiquitine-proteasome machinery by proteasome inhibitors may lead to cell destruction by promoting further aggregation of stabilized oncoviral proteins which may in turn perpetuate the activation of intracellular stress cascades and, consequently, it may lead to the eventual activation of proapoptotic signaling and tumor cell death.

### Molecular implications of EBV infection and proteasome alterations in survival and metastatic potential of NPC

Despite the high cure rates of NPC, local recurrence as well as development of distant metastasis are still the main causes of treatment failure and represent the main prognostic markers of this disease. In this sense, strong efforts are being developed so as to identify potential molecular markers implicated in the molecular pathogenesis of NPC-related metastasis and tumor recurrence.

S100 calcium-binding protein A14 (S100A14) is a member of the multi-functional S100 protein family. Constitutive expression of this protein has been found to be deregulated in several cancer types. To this matter, Meng et al. [41] showed that reduced expression of S100A14, which is mainly induced by promoter-methylation, was significantly associated with shorter Overall Survival (OS) and Disease Free Survival (DFS) in NPC samples. Further analysis elucidated that S100A14 plays a crucial role by inhibiting the NF-kB signaling pathway, thereby reversing the epithelial-mesenchymal transition (EMT), which may in turn promote an indirect suppression of cell proliferation and metastasis in NPC. In fact, functional assays confirmed that S100A14 suppressed NPC cellular migration by enabling the ubiquitin-proteasome-mediated degradation of interleukin-1 receptor-associated kinase 1 (IRAK1). To further describe the implication of this pathway, the authors found that pretreatment of S100A14-overexpressing cells with the proteasome inhibitor MG132 was able to recover the IRAK1 expression, suggesting that S100A14 facilitates the ubiquitination and proteasome-mediated IRAK1 degradation [63]. This molecular pathway is commonly shared among different neoplasms, as S100A14 upregulation happens in most cancer types aside from esophageal carcinoma, head and neck squamous cell carcinoma and skin cutaneous melanoma [64, 65].

On the other hand, cyclin-dependent kinase inhibitor p27 is widely known to be primarily regulated by the ubiquitin-proteasome pathway [66]. Pan et al. [42], described an interaction between Jab1/CSN5 and p27 in NPC cells, by which Jab1/CSN5 may control p27 degradation by direct targeting. In this sense, treatment of NPC cells with proteasome inhibitors resulted in a significant increase of total p27 levels. In parallel, Jab1/CSN5 overexpression happened to be inhibited, which may therefore indicate that Jab1/CSN5 promotes p27 degradation through the ubiquitin-dependent proteasome pathway which in turn may lead to cell-cycle progression [43].

Pharmacologic approach in tumor cell lines has shown different natural compounds and natural product derivatives with pharmacologic activity as suitable proteasome inhibitors [67]. Thus, curcumin is able to induce cell-cycle arrest of EBV-positive NPC cells thereby favoring further apoptosis. Furthermore, curcumin has also shown pharmacologic potential to promote EBNA1 degradation via proteasome-ubiquitin pathway which leads to further inhibition of cell proliferation [43]. Also, experimental fishing of target protein using bioinformatic approaches has identified up to 20 artemisinin-interacting proteins, some of them correlated with artemisinin-induced 26S proteasome subunit deregulation [68]. The anti-tumoral effects of artemisinin have been widely reviewed in the literature, providing data on its antiproliferative and immune response-modifying properties of tumor cells [69, 70]. In addition, combinatorial regimens represent an expanded strategy in cancer treatment. To this matter, both bortezomib as well as the hystone deacetylase inhibitor SAHA have proven to synergistically induce cellular apoptosis as well as inhibition of EBV replication in NPC cell lines. SAHA-based single pretreatment of NPC cell lines showed a significant increase of Zta (EBV immediate-early lytic protein) expression in EBV-positive NPC cells, whereas combination of SAHA with bortezomib was found to decrease the SAHA-induced Zta expression. Interestingly, the synergistic effect described with the combination of SAHA and bortezomib in terms of cellular apoptosis was similarly observed in both EBV-positive as well as in EBV-negative NPC cells [45]. Moreover, this effect was specifically found among NPC tumor cells and was not reproduced in normal nasopharyngeal cells. Further studies revealed an EBV lytic cycle-independent apoptosis. This effect was identified, both in vivo and in vitro, to be mainly exerted by sequential ROS (Reactive Oxygen Species) generation, ER (Endoplasmic Reticulum) stress induction and downstream activation of caspase 4 [44]. Thus, combinatorial regimens based on the aforementioned strategies may represent a dual therapeutic advantage by both, blocking EBV replication in NPC cells as well as by directly increasing tumor apoptosis [71].

Another pharmacologic approach studied in the setting of NPC is the use of molecules targeting the Inhibitor of Apoptosis Protein 2 (c-IAP2), as high concentrations of c-IAP2 have been identified in NPC cell lines, supporting the hypothesis that c-IAP2 may play a critical role in the molecular pathogenesis of NPC. To this matter, pharmacologic inhibition of c-IAP2 demonstrated a dramatic decrease of c-IAP2 levels within NPC cells. Further proteomics revealed markedly elevated levels of ubiquitinated c-IAP2 in NPC cell lines previously treated with c-IAP2 inhibitors. Actually, pretreatment of NPC cells with the proteasome inhibitor MG132 or epoxomicin showed to prevent c-IAP2 decrease after c-IAP2 inhibitor treatment. Thus, the authors conclude that pretreatment of NPC cells with c-IAP2 inhibitors may induce proteasome-dependent degradation of c-IAP2 by directly enhancing polyubiquitination of c-IAP2 substrate [44, 71, 72]. Interestingly, different studies have shown that bortezomib is able to upregulate the proapoptotic protein NOXA which is directly implicated in the negative interaction together with Bcl-2 and Bcl-XL, thereby favoring tumor cell apoptosis [73]. Main in vitro and in vivo studies are briefly summarized in Table 1.

### Host factors influencing NPC carcinogenesis

In addition to EBV infection and environmental exposures, there are other factors which are centrally involved in the pathogenesis of NPC [74, 75].Beyond activation of diverse protoocongenes, which plays a widely recognized role in the pathogenic development of cancer, both gene mutations as well as epigenetic alterations can extensively affect eventual cellular behavior. Genome wide sequencing, high density SNP analysis and detection of DNA methylation biomarkers, have allowed the in-depth study of the genome and, conclusively, a profound understanding of genes associated with oncogenesis. This fact has enabled the identification of tumor susceptibility, intrinsically prognostic subgroups, as well as tumor progression nature and therapeutic sensitivity. To this matter, prevention and early detection of cancer together with close monitoring of recurrence are of paramount importance in establishing prognosis and outcome of the disease and, globally, in determining novel therapeutic strategies. So on, proteasome dysregulation seems to play a role in a EBV independent maner. In this line, several chaperones involved in the proteasome-ubiquitin system, are responsible for posttranslational modifications and for the special folding of secreted proteins which occur in the endoplasmic reticulum (ER). As a consequence, ER related stress can be triggered by proteasome inhibition, inducing potential fatal proteotoxicity and finally the cell death [76–78]. For all of the above, the detection of new and reliable biomarkers is a clinical priority.

Among different strategies assessing novel biomarkers in NPC, missregulation of the NF-κB pathway, either by mutation or epigenetic mechanisms, is also involved in cancer pathogenesis. Zheng et al. [79],characterized the mutational landscape of 135 NPC samples using whole-exome sequencing and targeted re-sequencing. In this work, multiple loss-of-function mutations were identified in several NF-κB signaling negative regulators such as *NFKBIA*, *CYLD*, and *TNFAIP3*. Functional studies also confirmed that inhibition of NFKBIA has a significant impact on NF-kB activity and NPC cell growth. Moreover, an APOBEC (apolipoprotein BmRNA-editing enzyme, catalytic polypeptide-like)mediated signature was described. On the other hand, *CYLD* (cylindromatosis lysine 63 deubiquitinase), a NF-kB inhibitor, has been described as one of the most frequently mutated genes in NPC. Functional assays indicate that CYLD suppresses NPC tumorogenicity and metastasis by negatively regulating the NF-kB signaling pathway [80]. Consequently, the new era of cancer therapy, based on cancer genetics and genomics along with an improved knowledge on cancer hallmarks, has increased the interest of the therapeutic potential of targeting NF-kB in cancer [81].

In the line with the above mentioned, NESG1 (also known as CCDC19) has been described as a potential tumor suppressor in NPC. NESG1 is specifically expressed in the epithelial cells of nasopharynx and its reduced expression is an unfavorable factor which is related with further promotion of NPC progression and poor prognosis [82]. Interestingly, a-enolase (*ENO1*), which has been shown to be overexpressed in NPC [83] samples, has been described as a NESG1-regulated protein in NPC cells, given that NESG1 expression is negatively correlated with ENO1 expression in NPC tissues. Even more, overexpressed ENO1 has not only shown to restore cell proliferation and cell-cycle progression, but also to antagonize eventual regulation of NESG1 over other cell-cycle regulators such as p21 and CCNA1. Besides, higher expression of ENO1 has demonstrated to induce the expression of c-Myc, pRB, and E2F1 in NESG1-overexpressed NPC cells [84]. In this line, miR-1254 was confirmed as a positive downstream modulator of NESG1, further reducing the metastatic potential of NPC cells in vivo and in vitro. miR-1254 acts as an independent prognostic factor for NPC, which is induced by NESG1 to suppress NPC metastasis via inactivation of Wnt/b-catenin pathway and its downstream EMT signaling [85]. Thus, ectopic miRNA expression could display diverse antitumor effects offering potentially new therapeutic strategies.

In addition, the vacuolar protein sorting 33B (VPS33B) is rarely reported in malignant tumors. However, it has been shown that overexpression of VPS33B inhibits proliferation and bypasses chemoresistance to 5-fluorouracil in NPC. Moreover, VPS33B interacts with NESG1 by colocalizing in the cytoplasm. Literature so far has shown that knockdown of NESG1 is able to reverse the inhibitory effect of the overexpression of VPS33B in NPC cells by downregulating the PI3K/AKT/c-JUN-mediated transcription repression. Curiously, VPS33B is downregulated by viral LMP-1 and nicotine, and thus suppresses the proliferation of NPC cells by disrupting its interaction with NESG1 in NPC cells [86].

On the other hand, O6- methylguanine–DNA methyltransferase (MGMT) has been well-characterized to be a therapeutic determinant of O6-alkylguanine alkylating drugs, and it is related to cisplatin resistant in NPC models. Thus, it has been shown that platinum levels after cisplatin exposure is significantly lower in MGMT-proficient cells than in MGMT-deficient ones, and that MGMT protein directly binds to cisplatin-induced DNA damage. Proposed mechanisms suggest that cisplatin-bound MGMT protein became ubiquitinated and eventualy degraded through the proteasome-ubiquitin system. As a consequence, patients who received cisplatin treatment who did show higher MGMT expression level, did also exhibit shorter progression free survival and overall survival compared with patients with lower MGMT expression [87]. Therefore, quantification of MGMT levels may play a role for patients undergoing cisplatin-based therapy together with PIs, given the higher risk for cisplatin accumulation and the resultant toxicity.

Another interesting fact is that the expression of ANXA1 and EphA2 is significantly higher in NPC tissues than in normal nasopharyngeal epithelium. Basally, ANXA1 competes with Cbl for binding to EphA2, which in turn increases its stability by inhibiting Cbl-mediated EphA2 ubiquitination and degradation in NPC [88]. Feng et al. showed that disruption the ANXA1 and EphA2 complexes downregulates EphA2 expression further supressing NPC cell derived oncogenicity.

Additionally, TMEM8B-a protein, which represents an isoform of the NGX6 gene product (Transmembrane protein 8B) has been described as a metastasis suppressor in NPC. TMEM8B-a is rapidly degraded via proteasome pathway which is mediated by Ezrin in many NPC and lung cancer cell lines. However, TMEM8B-a is not ubiquitinated. To this matter, Wang et al. demonstrated that cell lines with stable TMEM8B-a protein expression treated with MG-132 or bortezomib could prevent TMEM8B-a degradation [89].

Following other factors involved in NPC carcinogenesis, the expression of CULLIN 3 is increased in both NPC tissues and cell lines, and correlate with Ki-67 based proliferation index in NPC samples. Moreover, in patients with NPC, the overexpression of this protein is associated with local relapse and distant metastasis. In vitro experiments show that knockdown of CULLIN 3 is related to a reduced proliferation rate, inhibition of tumor cell formation as well as to reduced migratory and invasive capacity of NPC cells. In addition, Zeng et al. also suggested that the CULLIN 3 based ubiquitin proteasome targeting may be a useful and promising therapeutic target in NPC [90].

As a matter of fact, polymorphisms in the genes of histocompatibility locus antigens (HLA) are reported in NPC. These polymorphism might play an important role given the resultant lack of proper immune functions against EBV infection and, eventually, for NPC perpetuation in endemic populations. Chin et al. describe a strong association of HLA-A with NPC susceptibility in a GWAS based study of Malaysian Chinese patients [91]. Furthermore, Chattopadhyay et al. showed a significant association between widely distributed microsatellites in three different HLA regions and the susceptibility of NPC development in a cohort of Northeast Indian population [92]. In fact, the HLA-E polymorphism in NPC pathogenesis and prognosis was deeply studied by Douik et al. in a Tunisian case-control study. Authors found that HLA-E*1031 and HLA-E*1032 variants were associated with NPC patients whereas HLA-E*1033 was mainly associated with healthy control [93]. Additionally, other case-control study conducted in the Northern Region of Portugal which assessed eight different single nucleotide polymorphisms revealed no statistically significant differences between genotype distributions in all of studied polymorphisms. However, the results for HCGA9 rs6457110 polymorphism showed a tendency for an increased risk of NPC development among carriers [94].

In summary, NPC carcinogenesis is a diverse and molecularly complex process in which, aside for EBV driven molecular alterations, many other EBV-independent environmental as well as molecular factors take part. Untangling these processes seems crucial for their potential implications in different clinical fields, both in prevention, diagnosis and tumor prognosis as well as in the therapeutic setting of NPC.

### The role of proteasome dysregulation in NPC related immunologic phenotype

Tumor microenvironment in NPC is highly defined by its immune-tolerant nature which leads to further avoidance of tumor cell recognition and, thus, to immune cell-mediated tumor excytotoxicity. Immunotherapy aims to promote cell to cell recognition by either inhibiting tumor mediated immunologic silencing or by enhancing immunologic recognition of tumoral cells. In this way, since immunotherapy strongly irrupted in Oncology, it has demonstrated promising results in different tumors and, specifically, in NPC [73, 95–97].

The central implication of EBV in natural pathogenesis of NPC plays a crucial role in terms of immunity and carcinogenesis. To this matter, innate immunity is important for viral recognition and primary host defense. To this matter, Retinoic acid-inducible gene I (*RIG-I*) as well as melanoma differentiation associated protein 5 (MDA5) are both cytosolic receptors implicated in the type 1 interferon (IFN-I) induced primary immune response which is responsible for viral recognition and destruction. Thus, RIG-I activation has been proposed as a feasible therapeutic strategy in diverse neoplasms [46, 98–100], among which NPC seems of highest interest given the role of EBV infection within further regulation of RIG-I expression in this disease [101]. Regarding this key interaction, Xu et al. found that LMP1 induced both, downregulation of Sendai virus infection, RIG-I degradation as well as further induction of IFN-β synthesis in murine models. Further studies revealed significantly lower levels of RIG-I in C666–1 cells (EBV positive NPC cells) compared to EBV negative NPC cell lines. In fact, treating cultures with INF-α did not yield differences in RIG-I expression. In addition, further testing using proteasome inhibitor MG132 was able to partially inhibit LMP1 mediated RIG-I degradation, for which LMP1 might be responsible for the recruitment of E3 ligases and, therefore, for proteasome-dependent RIG-I degradation in EBV positive NPC cells [101]. Moreover, RIG-I levels seem to be closely related to EBER transcriptional rate in NPC cells, highlighting the significant role of EBV infection in evading RIG-I production, and thus, in avoiding further immune recognition and cell death. Interestingly, these results have also been described in diverse RNA viruses as suggested by different authors [102–104]. As a matter of fact, expression of RIG-I has been extensively described in EBV associated tumors such as Burkitt’s lymphoma and Hodgkin lymphoma [105, 106]. In this scenario, authors conclude that EBV has developed unique strategies for evading innate immune response based on its unique viral machinery for which novel strategies are been developed with the aim of targeting virally induced immune exclusion.

On the other hand, IFN-γ is a key antitumor cytokine secreted mainly by activated Th1 and Natural Killer cells which has been shown to be significantly increased in Tumor Infiltrating Lymphocytes from NPC derived tumor stroma compared to healthy controls. In addition, INF-γ is a well known inductor of Indoleamine-2,3-dioxygenase (IDO) expression in diverse tumors, which is known to be a major contributor of tumor-induced immune exclusion [107] and, therefore, it may represent a promising target in NPC treatment. To this matter, different studies have investigated the role of proteasome inhibitors showing that bortezomib was able to decrease the expression levels of IDO in NPC cell lines as well as to increase tumor sensitivity to active immune recognition and immune mediated excytotoxicity. Moreover, bortezomib showed to actively inhibit STAT1 and to further activate IRF-1 pathways in NPC cells, which are responsible for activating the transcription of IDO and its further expression. Bortezomib exerts its pharmacologic mechanisms by attenuating IRF-1 degradation via proteasome inhibition, as well as by directly inhibiting STAT1 phosphorylation and its further translocation to nucleus which leads to inhibition of IDO expression [73]. In addition, bortezomib also promotes degradation of IkB-a which otherwise prevents NF-kB pathway activation that is further responsible for triggering an active immune response [108, 109]. Therefore, bortezomib and proteasome inhibitors may provide therapeutic benefit, especially in potentially active combinatorial strategies aimed to promote immune recognition and immune mediated cell death. Molecular implications on the use of all the aforementioned treatments are illustrated in Fig. 2.

As mentioned above, immunotherapy (IO) has become an essential among the oncologic therapeutic armamentarium proving to be effective in different tumor types among which NPC needs to be remarked. NPC is characterized by high expression of Programmed Death Ligand 1 (PD-L1) as well as by the abundant presence of Tumor Infiltrating Lymphocytes (TILs) which highlights the immunogenic nature of NPC which makes this disease highly suitable for therapies targeting the immune checkpoint blockade [110–113]. Moreover, some studies have identified viral LMP1 protein and IFN-γ as main inductors of PD-L1 expression in NPC cells [114]. In this sense, different clinical trials based on IO alone as well as in combinatorial regimens together with chemotherapy and IO have rendered promising results in terms of Progression Free Survival (PFS) and Overall Survival (OS) [115–117].

Altogether, given the immunogenic nature of NPC as well as its complex pathomolecular characteristics involving EBV infection and EBV-derived cellular alterations, which include proteasome alterations, combination of proteasome inhibitors together with IO may represent a promising therapeutic modality in NPC. Therefore, future studies are granted to elucidate the role of IO-based strategies as well as to study potential therapeutic combinations aimed to look for multi-targeting modalities that may favor tumor response by blocking tumor proliferation at different pathomolecular levels.

### Clinical experience with proteasome inhibitors in NPC

The use of proteasome inhibitors (PI) is an extended practice in different hematologic tumors and, to this date, several drugs have been developed and approved. The major clinical use of these inhibitors has been linked to multiple myeloma where the firstly approved peptide bortezomib boronate (Velcade®) had a great positive efficacy as a first-in-class agent for this disease. Also, second generation PI have been developed such as carfilzomib (FDA approval in 2012), marizomib, ixazomib (FDA approval in 2015), oprozomib and delanzomib with promising clinical outcomes. Their toxicity can vary from mild nausea and diarrhea up to a non-reversible peripheral neuropathy leading to drug withdrawal. Furthermore, the study of PI has been developed in other malignancies such as leukemia, lymphomas and solid tumors; even in the setting of GVHD (Graft-Versus-Host-Disease). For example, marizomib is currently being investigated in glioblastoma (NCT03345095), bortezomib plus vandetanib in solid tumors with special focus on medullary thyroid cancer (NCT00923247), bortezomib plus herceptin for advanced breast cancer with HER2 overexpression (NCT00199212) and orIxazomib and selinexor for sarcoma (NCT03880123) among others. A recent phase I trial evaluated the maximum tolerated dose, safety and preliminary efficacy of lapatinib plus bortezomib, in adult patients with refractory solid tumors [118]. Although no toxicities were reported, no tumor response was observed at the dose levels tested, and the study was closed early.

The novel VLX1570 small molecule which induces inhibition of deubiquitinases has been tested in multiple myeloma, but due to pulmonary toxicity, the study was discontinued [119]. However, efforts directed to identify deubiquitinase-inhibitors with greater therapeutic effect is warranted based on the unique mechanism of action, robustness of preclinical antitumor activity, and activity of the deubiquitinase inhibitors in PI resistant multiple myeloma targeting the 20S proteasome subunit.

As previously highlighted, Pis have been satisfactorily explored among different hematologic malignancies. However, the role of PIs in solid tumors remains under active research. In fact, therapeutic strategies exploring the role of PIs in solid tumors seem of highest interest given the conceptual application of these drugs among solid tumors. Up to now, lung cancer has been the most extensively studied tumor regarding the combinatorial use of PIs. For instance, preliminary clinical approaches using bortezomib as a single therapy in both platinum-refractory small cell lung cancer (SCLC) as well as in non-small cell lung cancer (NSCLC) failed to prove their efficacy. So on, the phase II trial studying the role of bortezomib as a single-agent based therapy in refractory SCLC failed to prove efficacy In this subset of patients with a mean PFS and OS of 1 month and 3 months respectively [120]. Moreover, single agent bortezomib failed to prove any further efficacy in the second line setting of NSCLC, neither in the KRAS G12D and TP53 deficient group with a median PFS of 1 month, median OS of 13 months and Overall Response Rate (ORR) of 6% of all patients [121]. Even if these results may appear disappointing, the really interesting point in the setting of the utility of bortezomib among solid tumors comes from combinatorial strategies. In this scenario, combination of bortezomib together with carboplatin, paclitaxel and radiotherapy in locally-advanced NSCLC patients showed surprisingly long survival results with a PFS of 76% unprogressed patients at 1 year follow-up together with a median OS of 25 months [122]. Even if this was a phase II trial without direct comparison of bortezomib including regimen with the bortezomib non-containing arm, these results are far from those documented in classic trials of platinum doublet combining regimens together with radiotherapy (the LUN 56 and LUN 63 trials [123]). As for the metastatic setting, combination of docetaxel together with bortezomib reported higher results in terms of PFS and OS as those previously reported with single-agent chemotherapy based trials in NSCLC [124], although no direct comparison has been performed. In parallel, addition of bortezomib to cisplatin have yielded promising results in terms of synergistic activity and eventual tumor regression in murine models of platinum refractory SCLC [125].

Conclusively, even if proteasome inhibition as single therapy has modestly proven to have no activity among certain solid tumors as in the case of lung cancer, we find highly interesting that the addition of bortezomib to classic chemotherapy as combinatorial or maintenance regimen seems to enhance antitumoral activity over classic approaches. To this matter, we aim to emphasize our idea that combinatorial strategies multi-targeting tumor pathobiology may show promising results in further controlling neoplastic disease. As a result, addition of PIs together with novel therapies, if not extensively studied so far, might add valuable benefit to patients with solid tumors in which proteasome dysregulation may play a role.

Following the previous point, and in regard of NPC, few clinical trials have tried to prove the efficacy of PIs in the clinical setting of recurrent or metastatic disease (RM-NPC). The phase II trial conducted by Gilbert J et al [126] evaluated the efficacy of combining bortezomib and irinotecan in the setting of recurrent or metastatic head and neck tumors, among which NPC accounted for a 8.6% of the total population. Even if scarce activity was proven in head and neck tumors showing no more than a 13% response rate, no further subanalysis for the subgroup composed of NPC was eventually evaluated. On the other hand, the NCT00305734 trial is a phase II trial evaluating the clinical efficacy of bortezomib in combination with gemcitabine in RM-NPC from which results are pending. Similarly, NCT00367718 aimed to assess the efficacy of bortezomib alone in RM-NPC. However, the trial was withdrawn without further results.

In this sense, even if the information on the efficacy and safety of PIs in NPC is far from being satisfactory, we think that, given the advances mentioned above on the evidence about the molecular implication of proteasome in NPC and benefit observed in different solid tumors, the use of PIs should be newly reconsidered due to different reasons. On the one hand, PIs might not be useful as single therapy based regimens, albeit, they could play a role in combination with other novel antitumor therapies such as immunotherapy given the immune molecular background associated with proteasome dysregulation and tumor immunephenotype. On the other hand, combinatorial strategies including different immunotherapy-based modalities together with classic chemotherapy could also represent suitable strategies in the setting of both locally advanced and metastatic NPC.

### Futures perspectives of proteasome targeting in cancer

In line with the above mentioned, since classic strategies that have endeavoured the use of PIs as single-agent based therapy have failed to prove its efficacy, the initial development and clinical success in some tumor types, such as multiple myeloma as well as for the promising results of bortezomib in combination with other therapies in solid tumors, different strategies are granted in further developing innovative targets against proteasome. Nevertheless, targeting proteasome as a therapeutic approach in cancer has currently yielded different barriers that need to be eventually overcome.

For instance, resistance mechanisms and toxicity profiles of first generations PIs have casted doubts on their usefulness. Despite this fact, current diagnostic strategies together with omic profiling are increasingly playing a key role in developing more precise therapies. Since cancer cells may eventually develop acquired resistance to PIs by different mechanisms, two are the main lines in novel targeting of proteasome inhibition. To this matter, research focused on design and synthesis of novel compounds seems of high interest. Beside novel therapies, implementation of combinatorial strategies together with novel therapeutic approaches such as cancer immune-landscape targeting drugs seems of greatest interest.

Regarding acquired resistance to proteasome inhibitors, multiple mechanisms have been reported so far including aberrant expression of ubiquitin proteasome system pathway components, induction of active extracellular drug efflux as well as activation of signaling cascades that avoid apoptosis, thus promoting further cell survival and tumor progression with central implication of eventual upregulation of the Unfolded Protein Response by direct induction of Endoplasmic Reticulum Stress [127–130]. Interestingly, recent advantages in regard of proteasome biology, have aimed to describe the immunoproteasome. During oxidative stress or in response to proinflammatory cytokines, cells produce specialized proteasome assemblies called immunoproteasomes, in which antigen-presenting cells have greater basal expression levels, enhancing ligand generation for MHC-I, and allowing for surveillance of CD8+ lymphocytes [131]. To this matter, inhibition of immunoproteasome has arised a promising target in autoinflammatory disorders by novel design of selective and potent immunoproteasome inhibitors together with combinatorial strategies including immune modulatory drugs [132]. However, even if such scenario might seem counterproductive in the oncospecific setting as indirect inhibition of immunoproteasome activity in vitro has demonstrated to downregulate CD8+ lymphocyte number as well as surface expression of MHC I [131], it is of highest interest because of two main aspects. First, downregulation of CD8+ lymphocytes and MHC I expression is hypothesized to render a direct sensitization to Natural Killer mediated immunotoxicity [131]vv. On the other, hand, as other author’s pointed out before, given the relatively constant intracellular content of proteasome, standard non-immune proteasome may constitutively provide steady quantities of peptide precursors for MHC I maturation [131]. Such considerations are of highest interest given the major combinatorial possibilities that may offer together with diverse immunotherapeutic compounds such as those targeting the Programmed cell Death 1 (antiPD-1) and the Programmed cell Death Ligand 1 (antiPD-L1) which directly activate CD8+ susceptibility against tumor cells as well as novel compounds targeting Natural Killer Cells ligands such as NKG2A targeting monalizumab which eventually enhances NK mediated excytotoxicity [133]. Given the interest in targeting proteasome inhibition, bypassing acquired resistance as well as for the potential role of inhibiting the immunoproteasome, novel compounds aiming to improve the effectiveness and specificity for such units has been extensively documented [134–136], as in the case of oprozomib (ONX0914).

Other resistance mechanisms described so far have been recently documented involving both mitochondrial dysfunction [137] as well as tumoral production of exosomes [138], both representing interesting points for their potential therapeutic suitability. In addition, given the central role of deubiquitinases in the acquired resistance to proteasome inhibitors, proof-of-concept studies have demonstrated that enzymes involved in the ubiquitin-proteasome system can be effectively targeted [139–141], which represents another promising target in dealing with PI related resistance. In fact, new approaches that exploit protein turnover, ubiquitination as well as deubiquitination cascades are also under active research which brings new hope to this field as they may represent effective targets in novel PI resistance models [142]. Actually, one of the main disadvantages yet pending to overcome is that derived from drug to drug interaction in the setting of PI based combinatorial regimens, which as emphasized before, might represent a promising strategy in cancer. In this sense, active research in the field of deubiquitinase signaling have prompted new alternatives. To this matter, copper complexes are found to be able to inhibit different components of the proteasome machinery, and are proposed to be one class of metal-based anticancer drugs with PI activity [143]. In addition to mediating oxidative damage, combination of Cu(II) to protein samples decreases the thermal stability of ubiquitin [144], further leading to eventual aggregates of spherical compounds of ubiquitin [145] which eventually has shown to directly inhibit proteasome activity in different tumor models and cancer cell lines [146]. However, different from bortezomib, some Cu complexes, such as CuET (bis(ET)-Cu(II) complex), induce a weak accumulation of hypoxia inducible factor 1, establishing a difference in substrate selection respect to current proteasome inhibitors [147]. Thus, a main challenge in the next future is to reveal precise mechanisms, specific substrate aspects, and structure activity relationship of proteasome inhibition by copper complexes, as well as their profile activity and side effects [148].

In the line with the aforementioned, and despite the lack of activity observed for single agent bortezomib-based clinical trials in solid tumors [120, 121], combinatorial strategies together with chemotherapy based regimens have shown potentially encouraging results in the setting of both locally advanced and metastatic solid tumors [122, 124, 125]. Consequently, addition of PIs to classic chemotherapy may bring a potential benefit in the setting of both local and advanced solid tumors. Even more interesting to this fact seems the recent irruption of immunotherapy to the field of Clinical Oncology. Current strategies aiming to directly inhibit immune checkpoints have yielded effective results and have become part of the daily armamentarium in the therapeutic approach of solid tumors. To this matter, antibodies targeting the Programmed Death 1 (PD-1), such as pembrolizumab and nivolumab, others targeting Programmed Death Ligand 1 (PD-L1), atezolizumab, avelumab as well as the Cytotoxic T-lymphocyte-associated protein 4 (CTLA-4) inhibiting antibody ipilimumab have demonstrated their effectiveness in terms of both overall survival (OS) and progression free survival (PFS) in different types of solid tumors among which lung cancer [149], bladder cancer [150], renal cancer [151], head and neck squamous cell carcinoma [152] and melanoma [153] are to be mentioned. As for nasopharyngeal carcinoma, different immune checkpoint inhibitors (ICPis) have demonstrated their efficacy in the metastatic setting of the disease. The KEYNOTE 028 trial demonstrated and Overall Response Rate (ORR) of 26% in previously pretreated patients with advanced NPC and a Disease Control Rate (DCR) of 52% together with a median PFS of 6.5 months [113]. Nivolumab also demonstrated preliminary efficacy with a median 1 year PFS and OS rates of 19 and 59% respectively as well as a ORR of 20% [114]. The antiPD-1 antibody camrelizumab has also shown effective targeting of NPC with an ORR of up to 34% and up to 91% when combined with classic chemotherapy regimens based on cisplatin and gemcitabine [115]. As previously mentioned, PIs, and specifically bortezomib, have demonstrated to attenuate IRF-1 mediated activity thereby favoring STAT1 inhibition and a substantial decrease of IDO enzyme synthesis. Moreover, it has also shown the ability of further activating NF-kB signaling [73, 108, 109]. These key points are closely related to tumor inflammation and therefore, to further enhancement of intratumoral immune response, making tumor cells susceptible to immune recognition by ICPi mediated effect. So on, combination of ICPi together with PIs might represent a novel and promising possibility to further enhance immune mediated tumor recognition.

In addition to the aforementioned, other therapeutic strategies targeting immune mediated tumor recognition are those derived from the administration of modified viruses with oncolytic activity which may further be combined with ICPis and from which it has been observed promising activity in the therapeutic strategy of solid tumors [154]. In fact, combination of bortezomib together with a modified oncolytic model of *reoviridae* have yielded promising results in multiple myeloma which furthermore is associated with cytokine release and proinflammatory changes that may further favor more effective combinations with ICPis [155].

Finally, another innovative strategy targeting different neoplasms is Adoptive Cell Therapy (ACT). Primary studies using cytotoxic T lymphocytes (CTLs) with specific affinity for EBV antigen recognition proved to be safe in terms of toxicity and to further induce LMP2 specific immune response [156]. Despite the limited sample size of 10 patients, efficacy results were also promising, achieving an ORR in up to 20% of the sample and a DCR of 60% [156]. To this matter, ongoing clinical trials are testing the use of CTLs in combination with classic chemotherapy to further elucidate their role in the treatment of advanced NPC [NCT02578641]. As for PIs, combinatorial strategies together with the aforementioned modalities may further be of general interest in the setting of maintenance treatment.

Altogether, futures perspectives in NPC are under active development. The role of PIs has proved to be limited in single-agent based strategies. However, since combinatorial strategies in solid tumors support the role of PIs for their potential capacity of further enhancing treatment effect, their use in this scenario seems a promising future strategy. So on, current novelties in clinical use of PIs include the development of new compounds with more specific ability of targeting proteasome and to eventually enable suitable bypass to tumor acquired resistance. In addition, recent advances in the clinical approach of NPC based on immunotherapy and immunotherapy based combinations, may grant future studies evaluating the efficacy of combinatorial regimens aiming to elucidate the role of PIs together with chemo and immunotherapy for their molecular implications whitin tumor immunephenotype and lymphocytic exclusion.

## Conclusions

Molecular pathogenesis of NPC is highly complex and biologically varied. EBV related latent infection plays a central role in tissue damage, cellular hyperplasia and malignant transformation. To this matter, proteasome dysregulation remains a poorly understood, albeit, crucial key pathogenic point of NPC which is responsible for direct modulation of intracellular signaling, thereby perpetuating cellular transformation and uncontrolled proliferation. Therefore, proteasome alterations may interact globally throughout different mechanisms affecting cellular proliferation, nuclear instability as well as immune exclusion.

In this scenario, proteasome inhibitors represent a therapeutic strategy that despite its lack of activity among solid tumors in the case of single-therapy based approaches, combination with classic regimens of chemotherapy has yielded promising results as both induction or maintenance treatment. So on, even if literature so far assessing the use of PIs in solid tumors is scarce, mainly given their lack of activity as monotherapy, combinatorial strategies have yielded promising results in recent studies. Therefore, proteasome inhibitors may act as key deregulators of tumor-proteome balance which in combination with other therapies may result highly useful.

To this matter, and given the efficacy of PIs in different hematologic tumors, future in clinics brings with it novel compounds aiming to further overcome tumor acquired secondary resistances to first generation PIs. But what is most interesting, given the central role of preoteasome inhibition in favoring immune exclusion and evasion, we hereby present novel perspectives in the therapeutic setting of nasopharyngeal carcinoma that may grant further research activity to elucidate the role of PIs in combination with novel immunotherapy-based modalities as well as the molecular rationale beyond it, that may further support such combinations.

## Data Availability

Not applicable.

## Abbreviations

ACT: Adoptive Cell Therapy
AP-1: Activator protein 1
Bcl-2: B-cell lymphoma 2
Bcl-XL: B-cell lymphoma-extra large
Ca^++^: Calcium
CF: Ubiquitin ligase substrate adaptor
c-IAP2: Inhibitor of Apoptosis Protein 2
c-IAP2i: Inhibitor of Apoptosis Protein 2 inhibitos
CSN5: Fifth subunit of COP9 signalosome
Csp4: Caspase 4
DCR: Disease Control Rate
EBER: EBV-encoded small RNAs
EBNA1: Epstein Barr nuclear antigen 1
EBV: Epstein Barr Virus
E3: Ubiquitin ligase complex
gB: EBV glycoprotein B
FBXO2: F-box only protein 2
ICPi: Immune Checkpoint inhibitor
Id1: Inhibitor of DNA binding/differentiation 1
IDO: Indoleamine-2,3-dioxygenase
IO: Immunotherapy
IRAK-1: Interleukin-1 receptor-associated kinase 1
IRF-1: Interferon regulatory factor 1
Jab1: C-Jun activation domain-binding protein-1
LMP1: Latent membrane protein 1
LMP2A: Latent membrane protein 2A
MDA5: Melanoma differentiation associated protein 5
MDM2: Mouse double minute 2 homolog
NF-kB: Nuclear factor kappa-light-chain-enhancer of activated B cells
ORR: Overall Response Rate
OS: Overall Survival
PD-L1: Programmed Death Ligand 1
PFS: Progression Free Survival
RIG-I: Retinoic acid-inducible gene I
ROS: Reactive oxygen Species
S100A14: S100 calcium-binding protein A14
SAHA: Suberoylanilide hydroxamic acid
SCF: SKP1/Cul1/F-box protein
STAT1: Signal transducer and activator of transcription 1
Zta: EBV immediate-early lytic protein
